# Losing cichlid fish biodiversity: genetic and morphological homogenization of tilapia following colonization by introduced species

**DOI:** 10.1007/s10592-018-1088-1

**Published:** 2018-07-18

**Authors:** Asilatu Shechonge, Benjamin P. Ngatunga, Rashid Tamatamah, Stephanie J. Bradbeer, Jack Harrington, Antonia G. P. Ford, George F. Turner, Martin J. Genner

**Affiliations:** 1 Department of Aquatic Sciences and Fisheries, University of Dar es Salaam, P.O. Box 35064, Dar es Salaam, Tanzania; 2 Tanzania Fisheries Research Institute (TAFIRI), P.O. Box 9750, Dar es Salaam, Tanzania; 3 School of Biological Sciences, University of Bristol, Life Sciences Building, 24 Tyndall Avenue, Bristol, BS8 1TQ UK; 4 Department of Life Sciences, Whitelands College, University of Roehampton, Holybourne Avenue, London, SW15 4JD UK; 5 School of Biological Sciences, Bangor University, Bangor, Gwynedd LL57 2UW UK

**Keywords:** Hybridization, Alien species, African freshwater fishes, Tilapia

## Abstract

Among the many negative impacts of invasive species, hybridization with indigenous species has increasingly become recognized as a major issue. However, relatively few studies have characterized the phenotypic outcomes of hybridization following biological invasions. Here we investigate the genetic and morphological consequences of stocking invasive tilapia species in two water bodies in central Tanzania. We sampled individuals from the Mindu Reservoir on the Ruvu river system, and at Kidatu on the Great Ruaha–Rufiji river system. We screened individuals at 16 microsatellite loci, and quantified morphology using geometric morphometrics and linear measurements. In both the Mindu and Kidatu systems, we identified evidence of hybridization between indigenous Wami tilapia (*Oreochromis urolepis*) and the introduced Nile tilapia (*Oreochromis niloticus*) or blue-spotted tilapia (*Oreochromis leucostictus*). At both sites, purebred individuals could largely be separated using geometric morphometric variables, with hybrids occupying a broad morphospace among the parental species. Our data demonstrate that the gene pools and phenotypic identity of the indigenous *O. urolepis* have been severely impacted by the stocking of the invasive species. Given the lack of evidence for clear commercial benefits from stocking invasive tilapia species in waters already populated by indigenous congenerics, we suggest further spread of introduced species should be undertaken with considerable caution.

**Electronic supplementary material:**

The online version of this article (10.1007/s10592-018-1088-1) contains supplementary material, which is available to authorized users.

## Introduction

Hybridization is a widespread phenomenon in nature (Olden et al. 2004). In the field of invasion biology, hybridization is generally considered a negative process for biodiversity, as it can lead to the erosion of unique genetic diversity (Todesco et al. 2016). Hybrids may possess novel traits that enhance their potential to have deleterious impacts on indigenous populations (Gaskin and Schaal 2002; Facon et al. 2005). In freshwaters, genetic or demographic swamping during hybridization is now considered a major driver of biodiversity loss, alongside habitat loss and pollution (Scribner et al. 2000; Perry et al. 2002).

In Africa, freshwater ecosystems are critically important for both biodiversity and food security, supporting capture fisheries of major significance for inland human populations (Vörösmarty et al. 2010; McIntrye et al. 2016; Lynch et al. 2016; Winemiller et al. 2016). However, many major capture fisheries in Africa are overexploited, leaving little capacity for the successful expansion of existing fisheries through technological innovations or increased effort (Welcomme et al. 2010). The pressing need to increase fish production to meet the demand from a growing human population has led to initiatives to develop inland aquaculture across Africa. To date, such initiatives tend to have been based on a handful of species, among the most prominent being Nile tilapia (*Oreochromis niloticus*). The species has a natural distribution in the Nile system and west Africa, but is now successfully used in aquaculture throughout tropical and subtropical regions (Deines et al. 2016). However, following deliberate introductions or accidental escapes, Nile tilapia is now naturalized in water bodies in many of the 140 countries where it is cultivated (Deines et al. 2016).

Nile tilapia has been widely hybridized with other tilapia species in captivity to generate novel strains, many of them fertile (Eknath and Hulata 2009). There is also evidence of extensive hybridization of Nile tilapia with multiple indigenous *Oreochromis* species in the natural environment, including *O. mossambicus* in South Africa (D’Amoto et al. 2007), *O. andersoni* and *O. macrochir* in Zambia (Deines et al. 2014) and *O. esculentus* in Kenya (Angienda et al. 2011).

Tanzania is a hotspot for natural diversity of the genus *Oreochromis*. Here, Nile tilapia is native only to the Lake Tanganyika catchment (Trewavas 1983), but has been widely distributed across the country for aquaculture and fishery enhancement (Genner et al. 2013; Bradbeer et al. 2018; Shechonge et al. 2018). It was initially introduced into Lake Victoria in the 1950s (Goudswaard et al. 2002), where it is now the dominant species in the tilapia fishery with estimated landings of 36,000 tonnes per annum in 2011 (Mkumbo and Marshall 2015). At the same time, populations of the endemic Lake Victoria *Oreochromis esculentus* and *O. variabilis* have declined dramatically, perhaps through competitive exclusion and/or hybridization (Goudswaard et al. 2002). Most of the farmed and stocked Nile tilapia in Tanzania appears to have been sourced from Lake Victoria, which likely explains why it has been accompanied by blue-spotted tilapia (*Oreochromis leucostictus*), a relatively small-bodied species native to the Nile system of Uganda below the Murchison cataracts, which became established in Lake Victoria at the same time as *O. niloticus* (Goudswaard et al. 2002).

**Fig. 1:**
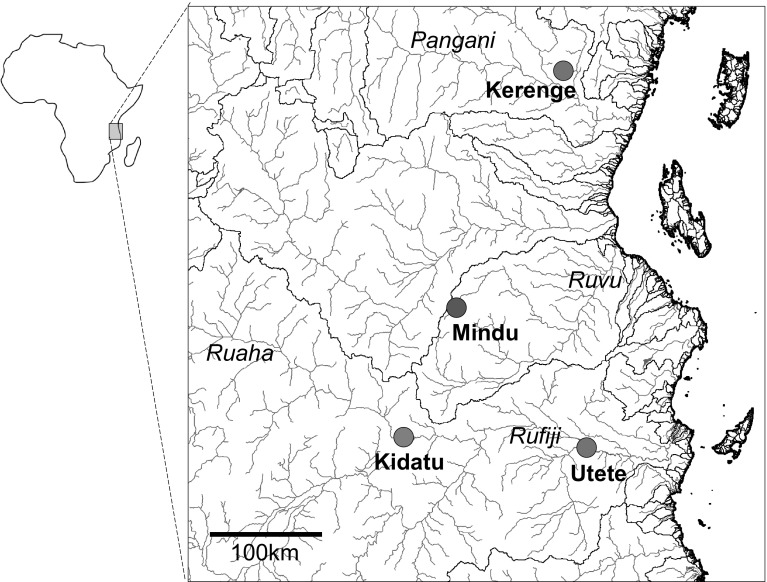
Sampling locations in Eastern Tanzania, including the focal sites (Mindu reservoir and Kidatu) and the sampling sites for reference material (Kerenge and Utete)

While sampling fishery catches in 2011–2012, we observed introduced *Oreochromis* in the Mindu Reservoir on the Wami river system (phenotypically *O. niloticus* and *O. leucostictus*) and at Kidatu on the Ruaha-Rufiji system (phenotypically *O. niloticus* only) (Fig. 1; Shechonge et al. 2018). In these areas, *Oreochromis urolepis* is the only indigenous *Oreochromis* species that has been recorded (Trewavas 1983; Eccles 1992). This large-bodied indigenous species continues to support major capture fisheries and represents a candidate species for future aquaculture, not least because of it tolerance of high salinity. In both the Mindu and Kidatu systems our field observations, based primarily on an apparent continuum of morphological traits and colouration of freshly landed individuals, suggested hybrids between *O. urolepis* and the introduced species were present. This was notable as there are no previous field-based records of hybridization between *O. urolepis* and either *O. niloticus* or *O. leucostictus*.

Here, we analyze specimens across the morphological range of individuals present in these systems to provide the first genetic tests of hybridization between these species in the wild, and to test if hybrids can be reliably distinguished on morphological characters alone. For the morphological work we chose to focus on (i) a combination of traditional linear morphological measurements of the head and body that are in principle readily measurable by fisheries researchers in the field, (ii) lower pharyngeal jaw (LPJ) measurements that can be indicative of dietary niche partitioning (e.g. Muschick et al. 2012), and (iii) geometric morphometric measurements of the head and body, that that can be powerful for discriminating cichlid species (e.g. Maderbacher et al. 2008).

## Methods

### Study sites

The study focussed on the two locations in Eastern Tanzania, the Mindu reservoir and the Kidatu reservoir. The Mindu reservoir is on the Ngerengere River, part of the Ruvu system, and construction of the dam began in 1983 and was completed in 1985. The reservoir maintains a water supply for the Morogoro region, and has a maximum depth of 12 m and a surface area of 5.1 km^2^ (Kashaigili 2011). The Kidatu dam is across the Great Ruaha river, part of the larger Ruaha/Rufiji/Kilombero system. Construction was completed in 1980, with a primary purpose in hydroelectric energy generation. The Kidatu reservoir has a maximum depth of 17 m and a surface area of 9.5 km^2^ (Yawson et al. 2006). At the time of sampling, both reservoirs supported small-scale artisanal fisheries activity.

### Sample collection and processing

Samples from the focal sites (Mindu and Kidatu) were purchased at the point of landing from artisanal fishers operating with gill nets. At both sites, we selected samples to cover the range of morphological variation and colour patterning present. At Kidatu, fish were sampled from both the reservoir behind the main dam, and from river beneath the dam (Table 1). The sampling sites at Kidatu were separated by approximately 12 km of river.

**Table 1:** Collection details and sample sizes for the genetic and morphometric analyses

Samples	Focal sites			Reference *O. leucostictus*	Reference *O. niloticus*	Reference *O. urolepis*
Location	Mindu reservoir	Kidatu reservoir	Kidatu river	Pangani river	Pangani river	Utete
Latitude	6.520°S	7.634°S	7.661°S	5.032°S	5.032°S	7.590°S
Longitude	37.360°E	36.885°E	36.972°E	38.548°S	38.548°S	38.450°E
Sampling dates	11–12 02/2015 25/02/2015	26/7/2015 17–19/9/2015 3/5/2016	3/5/2016	12/08/2015	12/08/2015	11/3/2015 29/4/2015
N genetics	158	94	25	9	30	49
N geometric morphometrics	148	94	25	–	–	–
SL (mm) geometrics (mean; range)	140 (85–226)	177 (120–265)	175 (149–234)	–	–	–
N body and head morphometrics	156	80	16	–	–	–
SL (mm) body and head (mean; range)	140 (85–226)	178 (122–265)	162 (151–184)	–	–	–
N pharyngeal jaw morphometrics	118	72	11	–	–	–
SL (mm) pharyngeal jaw (mean; range)	143 (85–226)	180 (122–265)	161 (151–184)			

Reference material was only used for genetic analyses

SL standard length (mm)

Samples from the reference species *O. niloticus* and *O. leucostictus* were collected from seine nets from Kerenge on the Pangani river, and thus allopatric to the indigenous *O. urolepis* (Table 1). The two species could be separated in the field on the basis of body shape and colour patterning, and at this site no hybridization between *O. niloticus* and *O. leucostictus* has been observed (Bradbeer et al. 2018). Reference (pure) samples of *O. urolepis* were collected from artisanal fishers at Lugongwe near Utete on the lower Rufiji river (Table 1). This site was chosen as no specimens of *O. niloticu*s or *O. leucostictus* have been collected in the vicinity during three sampling trips (August 2013; March 2015; April 2016), and therefore the population was inferred to comprise only purebred *O. urolepis*.

Individual fish were euthanised by anaesthetic overdose (MS-222 or clove oil), labelled and fin clips were taken and preserved in absolute ethanol for later genetic analysis. Fish were then pinned out on an expanded polystyrene (Styrofoam) board and allowed to dry so that body shape and fin structure were partially fixed, facilitating subsequent measurements. The labelled specimens were then immersed in 100% ethanol, before transfer to 70% ethanol for long-term storage.

### Microsatellite genotyping

DNA was extracted from fin tissue using the Wizard DNA extraction kit (Promega). Samples were initially screened at 18 microsatellite loci, sourced from Saju et al. (2010) and Liu et al. (2013) (Supplementary Information Table S1). PCR was performed in a volume of 10 µl consisting of 1 µl DNA (5 µl extracted DNA: 45 µl purite water), 5 µl Qiagen Multiplex 2x Mastermix and 4 µl primer mix (10 mM). Each primer was labelled with one dye from the DS-33 set (either 6-FAM, VIC, PET, NED). Loci were amplified within one of two multiplex PCR amplifications, each of which included one denaturation step of 15 min at 95 °C, followed by 35 cycles of 30 s at 94 °C, 90 s at 57 °C and 1 min at 72 °C, followed by a final extension step of 30 min at 60 °C. PCR products were then run on an ABI 3500 capillary sequencer with the LIZ 500 size standard, before being scored using GeneMapper 4.1 (Applied Biosystems, MA).

### Microsatellite data analysis

Estimation of genetic diversity, and associated tests of deviation from Hardy Weinberg Equilibrium, were made using default settings in Arlequin 3.5 (Excoffier and Lischer 2010). All individuals analyzed were screened using at least 13 of the 18 microsatellite loci. Two of the loci did not amplify successfully in all three species (Supplementary Information Table S2), and subsequent analyses were based on a core set of the remaining 16 loci that did amplify in all species (Supplementary Information Table S2). Genetic distances among specimens from both focal sites (Mindu and Kidatu) and reference samples, were ordinated using Factorial Correspondence Analysis in GENETIX 4.05 (Belkhir et al. 1999). Estimation of the genetic composition of individuals from the focal sites was achieved by first assigning individuals from both sites to one of three groups (K = 3) using the find.cluster function in the R package adegenet (Jombart and Ahmed 2011), selecting K = 3, and employing all principal components. This resulting classification was then used as a prior in Structure (Pritchard et al. 2000), using 10 runs. Each run was for 200,000 iterations, with the first 100,000 iterations discarded as burn-in. The output was then summarized in Clumpak (Kopelman et al. 2015). To illustrate the genetic composition of samples collected at the focal sites, excluding reference individuals, we again used Factorial Correspondence Analysis in GENETIX 4.05 (Belkhir et al. 1999).

To determine if our set of 16 loci were able to reliably identify hybrids, we simulated F1, F2 and backcross hybrids using HybridLab (Neilsen et al. 2006). We combined our purebred reference samples in a file with these simulated hybrids (100 individuals per hybrid category), and subjected them to the analysis procedure as described above using adegenet (Jombart and Ahmed 2011), and Structure (Pritchard et al. 2000).

### Geometric morphometrics

In the laboratory, the left-hand side of each preserved specimen was photographed with a scale bar. Images were loaded into tpsDIG 2.26 (Rohlf 2005) and landmarked with 25 landmarks (Supplemental Information Fig. S1), following Genner et al. (2007). At this point individuals with < 80% likelihood of assignment to one group from the Structure analysis were identified as potential hybrids and individuals with > 80% likelihood as potential purebreds. We then used MorphoJ 1.06 (Klingenberg 2011) to conduct a Procrustes Analysis on all individuals combining both sampling locations. The resultant Procrustes coordinates were then regressed against standard length, and the resultant size-standardised Procrustes residuals were used in a single Canonical Variate Analysis to visualize shape variation among groups. Discriminant Function Analysis (DFA) in SPSS v23 (Armonk, NY: IBM Corporation) was used to test for morphological differences among groups. We entered the Procrustes residuals as DFA predictor variables simultaneously, and assigned individuals to one of three “known” groups (potential purebreds), or an “unknown” group (all potential hybrids regardless of parental species). Discriminant Function axis scores for each individual were calculated for all individuals, including hybrids.

### Traditional measurements

We made 17 linear measurements of external morphology: standard length, body depth, head length, head width, inter-orbital eye width, snout length, lower jaw length, cheek depth, eye diameter, dorsal fin base length, anal fin base length, predorsal distance, preanal distance, prepelvic distance, preventral distance, caudal peduncle length and caudal peduncle depth. Additionally, we took four measurements from the lower pharyngeal jaw from calibrated images using tpsDIG 2.26 (Rohlf 2005): lower pharyngeal jaw length, lower pharyngeal jaw width, dentigerous area length and dentigerous area width. All measurements followed Snoeks (2004).

For each analysis of continuous data, measurements were log_10_ transformed, and isometrically size-standardized residuals were calculated for each variable using linear regression of each focal variable on standard length. Again samples from both sampling sites were pooled into the same analysis. Each set of size standardized residuals was entered into a Discriminant Function Analysis in SPSS v23, again entering the DFA predictor variables simultaneously, and assigning individuals to one of three “known” groups (potential purebreds), or an “unknown” group (potential hybrids). Discriminant Function axis scores for each individual were then calculated.

## Results

### Molecular identification of hybrids

**Fig. 2:**
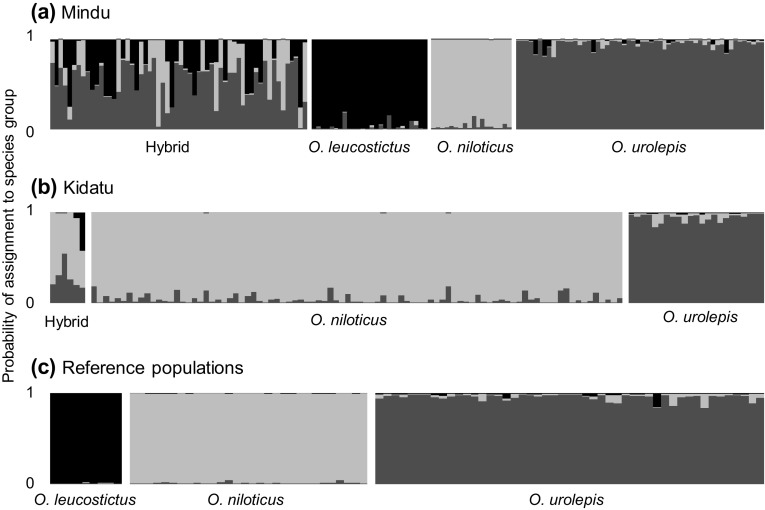
Results of Structure assignment to species groups (K = 3). Each row represents one individual fish. Individuals with > 0.80 probability of assignment of to a species group (80%) were assumed to be purebreds for subsequent analyses

Analysis of microsatellite allele frequencies indicated that our (purebred) reference material were assigned with greater than 80% probability, and so we used this as a threshold for the identification of purebred specimens from areas where hybridisation was suspected (Fig. 2; Supplementary Information Table S3). Our tests of simulated hybrids indicated that all F1 and F2 hybrids were correctly identified as hybrids using this 80% threshold, as were most (> 75%) backcross individuals (Supplementary Information Table S3).

**Fig. 3:**
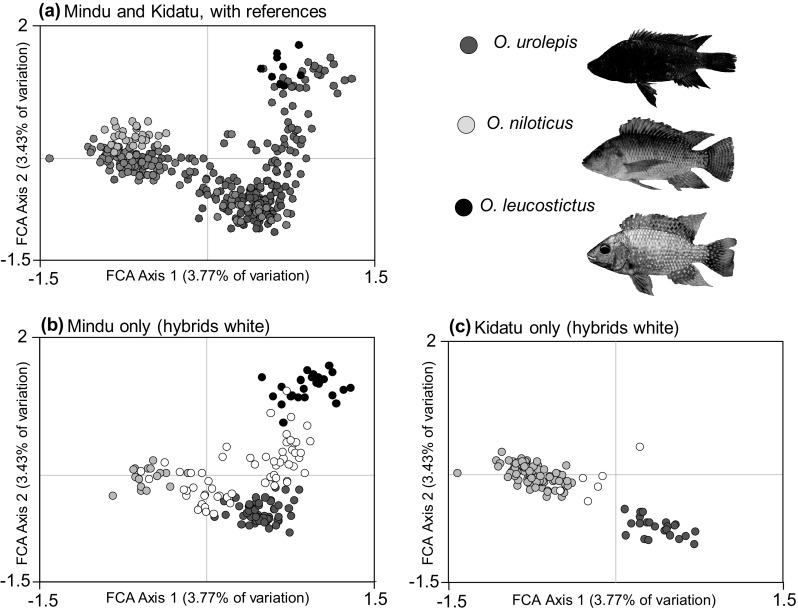
Factorial Correspondence Analysis (FCA) plots illustrating genetic similarity of sampled individuals. In **a** the FCA includes individuals from the Mindu Dam (blue) and Kidatu (grey), in relation to reference samples of *O. urolepis* (red), *O. niloticus* (yellow) and *O. leucostictus* (black). In **b** and **c** FCA plots from one analysis are presented for the Mindu and Kidatu sites respectively, with individuals assigned as putative purebreds (> 80% Structure assignment) as colored circles, and putative hybrids (< 80% Structure assignment) as white circles. (Color figure online)

**Fig. 4:**
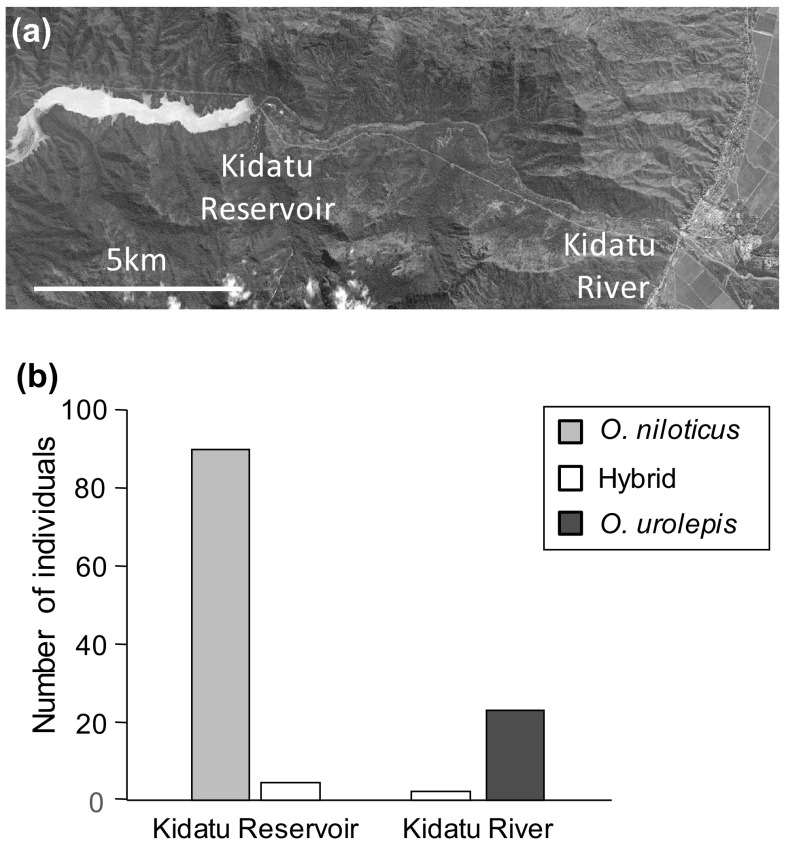
**a** Satellite imagery of the Kidatu region. Image data CNES/Airbus 13/7/2016 via Google Earth. **b** Number of individuals in the upstream reservoir and downstream river habitats sampled at Kidatu assigned to the purebred and hybrid genetic groupings

In this way, we estimated that our 158 genotyped individuals from the Mindu reservoir comprised 26 purebred *O. leucostictus*, 18 purebred *O. niloticus*, 56 purebred *O. urolepis*, and 58 individuals of hybrid ancestry. Of these admixed individuals, most were hybrids between non-native species and the native *O. urolepis* (Fig. 3). There were no clear-cut 1st generation hybrids between *O. niloticus* and *O. leucostictus*. At Kidatu, the 119 individuals genotyped were estimated to comprise 90 purebred *O. niloticus*, 23 purebred *O. urolepis*, and 6 hybrids. One individual from Kidatu was identified as possessing substantial (42.4%) *O. leucostictus* ancestry (possibly a 3-species hybrid: Fig. 2), while all others were largely *O. urolepis* × *O. niloticus*. At Kidatu there was spatial variation in the distributions of purebreds and hybrids. Individuals from the Kidatu reservoir were primarily purebred *O. niloticus* or *O. niloticus* × *urolepis* hybrids, while those downstream of the dam in river were primarily *O. urolepis*, with some *O. niloticus* x *urolepis* hybrids present (Fig. 4).

### Geometric morphometric analyses

**Fig. 5:**
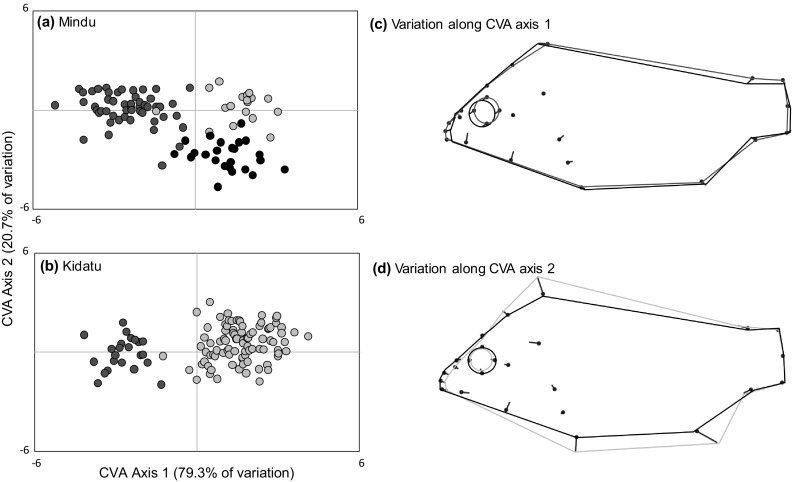
Canonical variate analysis illustrating differences in geometric morphology among individuals classified as purebred in samples from **a** Mindu and **b** Kidatu. Colours indicate *O. urolepis* (red), *O. niloticus* (yellow) and *O. leucostictus* (black). **c** and **d** Variation along axes in head and body shape, with coloured lines representing those species at the extremes of the distributions. Samples from both sites were pooled into the same analysis, but locations are shown separately. (Color figure online)

**Fig. 6:**
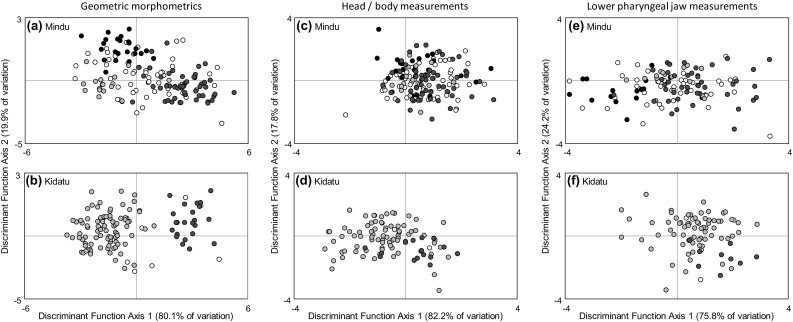
Results of Discriminant Function Analyses of **a, b** geometric morphometric and **c**–**f** traditional linear measurement data, with putatively purebred individuals (> 80% assignment to a species group), and putative hybrid individual (< 80% assignment to a species group). Colors indicate *O. urolepis* (red), *O. niloticus* (yellow), *O. leucostictus* (black) and hybrid (white). Samples from both sites were pooled into the same analysis, but locations are shown separately. (Color figure online)

Canonical variate analysis of geometric morphometric data based on individuals from the two admixture sites demonstrated substantial differences between the three species. Major axes of variation related to body depth and head shape. Canonical Variate axis 1 separated *O. urolepis* from *O. niloticus*, with *O. urolepis* possessing a longer snout and marginally reduced body depth relative to *O. niloticus*. Canonical Variate axis 2 separated *O. urolepis* and *O. niloticus* from *O. leucostictus*, with *O. urolepis* and *O. niloticus* having smaller eyes and greater body depths than *O. leucostictus* (Fig. 5). Discriminant function (DF) analysis revealed it was possible to significantly discriminate among populations on the first two axes (DF axis 1, Wilk’s λ = 0.101, χ^2^ = 417.658, df = 90, *P* < 0.001; DF Axis 2, Wilk’s λ = 0.502, χ^2^ = 125.386, df = 44, *P* < 0.001). The method was reliably able to separate and categorize purebred individuals, with 92.3% of individuals correctly classified (Fig. 6; Table 2). Discriminant function scores demonstrated that individuals of hybrid ancestry at both sites encompassed a broad geometric morphospace, with some individuals similar to parental species, and others intermediate (Fig. 6).

**Table 2:** Discriminant function classification results, combined across the two sampling sites

Genetic grouping	Predicted grouping from morphology			Total
	*O. urolepis*	*O. niloticus*	*O. leucostictus*	
Geometric morphometrics				
*O. urolepis*	69 (92.0%)	2 (2.7%)	4 (5.3%)	75
*O. niloticus*	2 (1.9%)	99 (91.7%)	7 (6.5%)	108
*O. leucostictus*	0 (0%)	1 (4.2%)	23 (95.8%)	24
Hybrid	27 (45.0%)	17 (28.3%)	16 (26.7%)	60
Head/body measurements				
*O. urolepis*	44 (63.8%)	8 (11.6%)	17 (24.6%)	69
*O. niloticus*	16 (16.8%)	60 (63.2%)	19 (20.0%)	95
*O. leucostictus*	6 (23.1%)	4 (15.4%)	16 (61.5%)	26
Hybrid	28 (45.2%)	17 (27.4%)	17 (27.4%)	62
LPJ measurements				
*O. urolepis*	33 (62.3%)	18 (34.0%)	2 (3.8%)	53
*O. niloticus*	22 (25.9%)	53 (62.4%)	10 (11.8%)	85
*O. leucostictus*	1 (6.3%)	2 (12.5%)	13 (81.3%)	16
Hybrid	18 (38.3%)	15 (31.9%)	14 (29.8%)	47

### Traditional morphological analyses

Traditional morphometric analysis could also discriminate among populations on the first axis (DF Axis 1, Wilk’s λ = 0.653, χ^2^ = 76.432, d.f. = 32, *P* < 0.001), but not the second (DF Axis 2; Wilk’s λ = 0.919, χ^2^ = 15.194, d.f. = 15, *P* = 0.438). The strongest correlates of variation along DF Axis 1 (Table 3), were indicative of *O. urolepis* having relatively longer snouts and longer heads relative to *O. niloticus* and *O. leucostictus* (Table 3; Fig. 6). The method was able to reliably classify only 63.2% of purebred individuals (Table 2), with hybrid individuals overlapping substantially with purebreds (Fig. 6).

**Table 3:** Correlations of morphometric variables with Discriminant Function Anaysis (DFA) axes

Trait category	Trait	DFA1	DFA2
Head and body	Anal fin base	0.339	0.076
	Body depth	0.082	− 0.500
	Cheek depth	0.398	0.316
	Caudal peduncle depth	0.169	− 0.006
	Caudal peduncle length	0.187	− 0.323
	Dorsal fin base length	− 0.170	0.168
	Eye depth	0.516	− 0.385
	Head length	**0.632**	− 0.027
	Head width	0.351	− 0.32
	Inter-orbital eye width	0.280	0.201
	Lower jaw length	0.194	0.204
	Preanal distance	− 0.190	0.039
	Predorsal distance	0.340	− 0.137
	Prepectoral distance	0.188	0.048
	Prepelvic distance	0.084	− 0.220
	Snout length	**0.772**	0.170
Lower pharyngeal jaw	Dentigerous area length	**0.600**	**0.775**
	Dentigerous area width	**0.947**	0.037
	Lower pharyngeal jaw length	**0.551**	0.274
	Lower pharyngeal jaw width	**0.738**	0.219

See Fig. 6 for the scores of individuals fish along these axes. Bold indicates the traits with the strongest associations with the DFA axes (> 0.6)

Discriminant function analysis of the lower pharyngeal jaw measurement data discriminated among populations on both of the first two major axes (DF Axis 1, Wilk’s λ = 0.553, χ^2^ = 88.538, d.f. = 8, *P* < 0.001; DF Axis 2, Wilk’s λ = 0.853, χ^2^ = 23.810, d.f. = 3, P < 0.001). DF Axis 1 was indicative of *O. urolepis* having on average the longest and widest jaw of the three species, *O. leucostictus* having the shortest and narrowest jaw of the three species, with *O. niloticus* intermediate (Table 3; Fig. 6). Again, this analysis was a weaker discriminator that the geometric morphometric analysis of body shape, being able to classify only 64.3% of purebred individuals (Table 2), while hybrid individuals occupied most of the morphospace of the purebred fish (Fig. 6).

## Discussion

Our molecular analyses supported our field observations that *Oreochromis* communities in the Kidatu and Mindu systems were comprised of multiple species, with reduced levels of heterozygosity across most loci consistent with the presence of genetic structure within populations (Supplementary Information Table S2). Further analyses were consistent with the presence of hybrids between the indigenous *O. urolepis* and the introduced *O. niloticus* and *O. leucostictus*. Evidence of hybridization between *O. niloticus* and *O. urolepis* has previously been demonstrated in aquaculture (Mapenzi and Mmochi 2016; Mbiru et al. 2016), but to our knowledge this the first evidence outside of captivity, and there are no previous reports of hybridization between *O. urolepis* and *O. leucostictus*.

Intriguingly, although we found *O. niloticus* × *urolepis* and *O. leucostictus* × *urolepis* hybrids to be commonplace in the Mindu reservoir, *O. niloticus* × *leucostictus* hybrids were less commonplace: indeed there was no clear-cut evidence of 1st generation hybrids between them. *Oreochromis niloticus* and *O. leucostictus* co-occur in Lakes Edward, George and Albert (Trewavas 1983), and thus it is plausible they are more strongly reproductively isolated than species pairs that are naturally allopatric in their distributions. Likewise, there is no evidence of admixture between these species at other locations in Tanzania that have been studied, including Lake Malimbe in the Victoria catchment, and Kerenge in the Pangani river system (Bradbeer et al. 2018). However, low levels of hybridization between *O. niloticus* and *O. leucostictus* have been reported in Kenya, but these involve the unique endemic subspecies *O. niloticus baringoensis* and specialized hot spring populations (Nyingi and Agnèse 2007; Ndiwa et al. 2014) likely to be genetically divergent from the Lake Albert/Nilotic populations of *O. niloticus eduardiensis* stocked in Lake Victoria and subsequently transferred around Tanzania.

To our knowledge this is the first evidence for substantive hybridization among introduced and indigenous *Oreochromis* in Tanzania. However, *O. niloticus* has been reported to hybridize with indigenous species elsewhere in Africa, including with *O. andersonii* and *O. macrochir* in the Kafue river in Zambia (Deines et al. 2014). When hybrids are viable and fertile, continued introgression into both parental species can occur, generating a hybrid swarm, and threatening the integrity of the indigenous species through genetic swamping (Todesco et al. 2016). Our data suggest that hybrids are viable, and that the complex patterns in the Structure plots indicates that some of the hybrids are probably backcrosses, indicating a hybrid swarm. Follow up analyses with larger numbers of nuclear markers would be useful to identify these backcrossed hybrids more accurately, and the extent and direction of introgression among the species. At present it is not known if the hybrids have reduced fitness relative to purebreds in either of the systems, but this may account for the continued presence of apparently purebred individuals at both locations. Postzygotic selection against hybrid individuals has been shown to contribute to species integrity in other fish communities including sticklebacks (Vamosi and Schluter 1999; Gow et al. 2007) and cyprinids (Nilsson et al. 2017).

A key component of ecological selection against hybrids would be some evidence of niche differentiation among the purebreds. Further work is required to establish if such differences are present in our study systems. However, *O. niloticus* and *O. leucostictus* have been noted as having divergent habitat preferences in the native Lake Edward, with *O. leucostictus* being most abundant in peripheral habitats, while *O. niloticus* is primarily found in open waters (Trewavas 1983). Similar niche differentiation is reported for these species in Lake Victoria (Seehausen 1996). It is plausible that divergent habitat preferences may also explain the dominance of *O. niloticus* in the reservoir at Kidatu, in contrast to the dominance of *O. urolepis* in the river downstream of the dam, although this could also be explained by deliberate stocking of the reservoir with *O. niloticus* and relatively poor survival of fish passing through the dam.

### Hybrid identification

Our field identification of purebred reference samples was based primarily on aspects of body colour, as all three species are distinguishable based on male breeding colors and patterning on the flanks and fins. Our study also demonstrated that purebred species were most readily separable using geometric morphometric characters, related to body depth and eye size, but linear measurements of head and body characters, and measurements of lower pharyngeal jaw morphology, also showed significance differences among the species. These results have relevance as they demonstrate that geometric characters can be used to assign individuals to species with reasonable confidence, and given close links between morphology and ecology in cichlids, then the species may differ in resource use patterns.

However, we discovered that hybrid individuals typically possessed a range of morphologies that were both overlapping with purebreds, and intermediate between them. We also found cases of hybrid individuals possessing morphologies that transgressed beyond those of the parental species. Such patterns are expected in cases where multiple hybrid generations are present, and novel combinations of alleles form (Seehausen 2004; Nichols et al. 2015). Since our results indicate that assignment of individuals based on morphology alone may lead to misassignment, we suggest that such classification should be coupled with evidence from genetic markers. It is also possible that colour patterning may be useful for hybrid identification, although this remains to be quantified, and may vary substantially between individuals depending on sex and reproductive state.

### Conservation

Our analyses provide the first evidence of extensive hybridization between indigenous and invasive tilapiine cichlids in Tanzania, and illustrate the potential vulnerability of Tanzania’s indigenous biodiversity to the threat posed by invasives. Here, the conservation of indigenous species faced by threats of hybridization with invasive species depends on the distribution of the invading species and the extent of hybridization. We have found *O. niloticus* and *O. leucostictus* to be distributed across multiple catchments in Tanzania, and to co-occur with *O. urolepis* in the Ruvu and Rufiji–Ruaha catchments as reported here, but we have also reported feral populations of *O. niloticus* and *O. leucostictus* in other locations where *O. urolepis* is indigenous. These include the Wami catchment (*O. leucostictus* and *O. niloticus*), the Mbwenkuru catchment (*O. niloticus*), as well as on Zanzibar (*O. niloticus*) (Shechonge et al. 2018). Further research is required to determine if hybrids are present in these systems, but the conservation of *O. urolepis* will likely depend on the identification and maintenance of habitats where *O. leucostictus* and *O. niloticus* are absent, and ideally these should be isolated from systems that harbour invasives. In North America, the concept of watershed-scale indigenous fish conservation areas is receiving increased attention (Williams et al. 2011), and the approach could be applied in Tanzania given there are still many smaller catchments, or parts of larger catchments, where invasive species have not been reported. For example, our genetic data support morphological observations suggesting an absence of invasives at Utete, and ox-bow lakes in the lower Rufiji region that contain only *O. urolepis* could be useful locations for conservation prioritization. Among other habitats in the region containing only the indigenous *O. urolepis* is Lake Mansi, an endorheic system between the Wami and Ruaha systems (Shechonge et al. 2018). Preservation of the biodiversity of these smaller catchments may benefit from formal recognition of their value for biodiversity conservation and potential for aquaculture future aquaculture.

## Concluding remarks

In summary, our work adds to a growing body of evidence that introduction of non-native fish species can lead to hybridization with indigenous species and threaten unique biodiversity. Such introductions are typically associated with attempts at capture fisheries improvement, or the use of novel aquaculture species in circumstances where has been insufficient biocontrol to prevent them from unintentionally entering nearby water bodies. With increased demand for fish protein, governments will need to manage the potential ecological impacts of aquaculture and initiatives to enhance capture fisheries. It has been proposed that zoned aquaculture should be established, where the only species used are indigenous to the location (Lind et al. 2012). At present there is no evidence that *O. niloticus* can lead to greater yield than large-bodied indigenous species, and indeed if *O. niloticus* stocks are contaminated with small-bodied *O. leucostictus* then yields may even decline. It may be useful to adopt the precautionary principle of avoiding introduced species, unless there is compelling evidence for their ability to improve fisheries without biodiversity loss. Practically, the implementation of effective biosecurity measures will require training of fisheries extension officers on the impact of introducing non-native species to natural water bodies, as well as building capacity in species identification.

## Electronic supplementary material

Below is the link to the electronic supplementary material.

Supplementary material 1 (DOCX 59 KB)

## Data Availability

Genetic and morphometric data analysed for this manuscript have been deposited Dryad (10.5061/dryad.38c4h63).
