# Supplementary material for: Losing cichlid fish biodiversity: genetic and morphological homogenization of tilapia following colonization by introduced species
Source: Conserv Genet. 2018 Jul 18;19(5):1199–209. doi: 10.1007/s10592-018-1088-1 (PMC6182432; doi:10.1007/s10592-018-1088-1)
Supplement: Supplementary file 1 — Supplementary material 1 (DOCX 59 KB) [file 10592_2018_1088_MOESM1_ESM.docx]

**Shechonge et al. Losing cichlid fish biodiversity: genetic and morphological homogenization of tilapia.**

**Supplementary Information Table 1**. Primer sequences and source publications of microsatellite loci used in the study.

| Marker name | Genbank accession | Primer sequence (forward) | Primer sequence (reverse) | Motif | Source |
| --- | --- | --- | --- | --- | --- |
|  |  |  |  |  |  |
| OM-01 | GU391020 | TTTAAAGTTACACAGCAGTACAAAG | TTGTAGCATTTCAACACAGTCTC | (GT)20 | Saju *et al.* 2010 |
| OM-03 | GU391022 | CTTTTTAATGAGCAACTTTTAAGTC | TGTGAATTTGACAACTTCCTTTC | (GATA)47 | Saju *et al.* 2010 |
| OM-04 | GU391022 | AGCTCAAAACCTCATACAAAGG | GCAGAGATGTCAGATGTTGTTC | (GACA)6 (GATA)16 | Saju *et al.* 2010 |
| OM-09 | GU391028 | GGCTACAACACCTGGATGG | TTGGGCTTACTGAAGCTGAC | (GT)26 | Saju *et al.* 2010 |
| OMO043 | JX204857 | GGGGTCATTCGGTTTATTGGTTAT | AGGGCAGGTCACGGGTTCG | (TTTG)8 | Liu *et al.* 2013 |
| OMO093 | JX204891 | AAGCCCCACATAGACGACCAGAGA | CAGAAACGGTGCCTGTTCCAGAA | (CAT)8 | Liu *et al.* 2013 |
| OMO100 | JX204895 | CCTTCCCCACCACTACCCTCATAA | CCCGCCCACACCTGACGA | (ATT)18 | Liu *et al.* 2013 |
| OMO114 | JX204905 | ACGCCTTAATGCTGCCTTCAAGA | TGATGCTCACCCCGTTCCTCA | (GTT)11 | Liu *et al.* 2013 |
| OMO129 | JX204914 | TTGGCAGGCTAAGTACTATTTCAT | GAGCGAATGGTTGTCTGTCTCT | (CCAT)9 | Liu *et al.* 2013 |
| OMO161 | JX204924 | ACTTTGACAAAAGAAGTGTAACAA | AGGGGAGGAGAAAATAAACTGTAT | (TAA)10 | Liu *et al.* 2013 |
| OMO219 | JX204964 | ATCCCCTTCTTTCCATCCCTGTC | AAGGCCTCTGTGAGCTGATTGATT | (TTTTG)10 | Liu *et al.* 2013 |
| OMO229 | JX204973 | GCGACTTTTTCTTTGCACATTTTT | AACTGAACCGCCATCATAATCATC | (GTT)9 | Liu *et al.* 2013 |
| OMO248 | JX204987 | AAAGACACAAAGAGAAACTAATCA | GGATGAATATTTAAAATCAGTCAG | (TCA)9 | Liu *et al.* 2013 |
| OMO337 | JX205052 | TAGGAGAGGCATAGGTTGTCAAAT | CAAGAGTCTAGGAGGGAATCAAAA | (GTTT)7 | Liu *et al.* 2013 |
| OMO361 | JX205069 | TGACAGCGAGCCAGAATGGAAGTA | AAAAGTGAAAGGGGCACAGTGAGG | (CTT)17 | Liu *et al.* 2013 |
| OMO391 | GR699257 | AGACATCTGTACGCTCTTTACGAA | AGTGCTAGAGGGAAGGGGCTGTA | (GAT)9 | Liu *et al.* 2013 |
| OMO392 | GR698887 | CTGGCTTAACTTCTCTACTGGACA | TCTACTCAAAACTGGCAACAAAAC | (GAATA)7 | Liu *et al.* 2013 |
| OMO397 | GR693794 | ACGCGTGTTTGAGATATTTAGATT | GAACAAACAAGGGGAGTGG | (GATT)7 | Liu *et al.* 2013 |

**Supplementary Information Table 2.** Genetic diversity of markers from sampled populations in this study. NA = number of alleles, HO = observed heterozygosity, HE = expected heterozygosity, P = probability of Hardy Weinberg Equilibrium.

| **Locus** |  | **Focal sites** | | **Reference populaions** | | |
| --- | --- | --- | --- | --- | --- | --- |
|  |  | Mindu | Kidatu | *O. leucostictus* | *O. niloticus* | *O. urolepis* |
|  | N ind | 158 | 119 | 9 | 30 | 49 |
| OM-01 | NA | 23 | 30 | 1 | 13 | 22 |
|  | HO | 0.506 | 0.681 | Monomorphic | 0.600 | 0.776 |
|  | HE | 0.727 | 0.913 | Monomorphic | 0.913 | 0.902 |
|  | P | < 0.001 | < 0.001 | Monomorphic | 0.000 | 0.013 |
| OM-03 | NA | 22 | 19 | 4 | 10 | 18 |
|  | HO | 0.506 | 0.681 | 0.556 | 0.700 | 0.367 |
|  | HE | 0.879 | 0.886 | 0.725 | 0.764 | 0.923 |
|  | P | < 0.001 | < 0.001 | 0.040 | 0.778 | < 0.001 |
| OM-04 | NA | 24 | 25 | 5 | 5 | 24 |
|  | HO | 0.304 | 0.370 | 0.889 | 0.167 | 0.673 |
|  | HE | 0.821 | 0.766 | 0.706 | 0.495 | 0.928 |
|  | P | < 0.001 | < 0.001 | 0.190 | < 0.001 | < 0.001 |
| OM-09 | NA | 27 | 27 | 1 | 8 | 24 |
|  | HO | 0.646 | 0.630 | Monomorphic | 0.767 | 0.776 |
|  | HE | 0.921 | 0.893 | Monomorphic | 0.602 | 0.912 |
|  | P | < 0.001 | < 0.001 | Monomorphic | 0.972 | 0.002 |
| OMO043 | NA | 10 | 8 | 5 | 5 | 5 |
|  | HO | 0.399 | 0.412 | 0.556 | 0.367 | 0.286 |
|  | HE | 0.767 | 0.777 | 0.719 | 0.328 | 0.709 |
|  | P | < 0.001 | < 0.001 | 0.179 | 1.000 | 0.000 |
| OMO100 | NA | 7 | 7 | 2 | 3 | 4 |
|  | HO | 0.342 | 0.429 | 0.444 | 0.633 | 0.082 |
|  | HE | 0.711 | 0.786 | 0.523 | 0.671 | 0.497 |
|  | P | < 0.001 | < 0.001 | 1.000 | 1.000 | 0.000 |
| OMO248 | NA | 11 | 13 | - | 7 | 12 |
|  | HO | 0.468 | 0.740 | - | 0.767 | 0.918 |
|  | HE | 0.800 | 0.843 | - | 0.762 | 0.902 |
|  | P | < 0.001 | < 0.001 | - | 0.055 | 0.766 |
| OMO093 | NA | 10 | 10 | 2 | 6 | 11 |
|  | HO | 0.627 | 0.664 | 0.111 | 0.867 | 0.878 |
|  | HE | 0.853 | 0.827 | 0.111 | 0.781 | 0.873 |
|  | P | < 0.001 | < 0.001 | 1.000 | 0.366 | 0.030 |
| OMO114 | NA | 8 | 6 | 2 | 3 | 4 |
|  | HO | 0.361 | 0.345 | 0.333 | 0.633 | 0.122 |
|  | HE | 0.734 | 0.728 | 0.294 | 0.660 | 0.596 |
|  | P | < 0.001 | < 0.001 | 1.000 | 0.102 | 0.000 |
| OMO116 | NA | 16 | 18 | 3 | 6 | 5 |
|  | HO | 0.709 | 0.765 | 0.889 | 0.533 | 0.204 |
|  | HE | 0.796 | 0.889 | 0.627 | 0.686 | 0.243 |
|  | P | < 0.001 | < 0.001 | 0.185 | 0.012 | 0.231 |
| OMO129 | NA | 9 | 8 | 3 | 3 | 8 |
|  | HO | 0.532 | 0.588 | 0.333 | 0.567 | 0.714 |
|  | HE | 0.817 | 0.799 | 0.464 | 0.635 | 0.760 |
|  | P | < 0.001 | < 0.001 | 0.106 | 0.536 | 0.635 |
| OMO219 | NA | 8 | 10 | 2 | 7 | 9 |
|  | HO | 0.475 | 0.639 | 0.111 | 0.667 | 0.755 |
|  | HE | 0.574 | 0.777 | 0.111 | 0.676 | 0.807 |
|  | P | < 0.001 | < 0.001 | 1.000 | 0.901 | 0.376 |
| OMO229 | NA | 7 | 9 | 3 | 3 | 8 |
|  | HO | 0.506 | 0.597 | 0.556 | 0.667 | 0.469 |
|  | HE | 0.769 | 0.748 | 0.523 | 0.621 | 0.782 |
|  | P | < 0.001 | < 0.001 | 1.000 | 0.176 | 0.000 |
| OMO337 | NA | 9 | 7 | 2 | 2 | 7 |
|  | HO | 0.468 | 0.429 | 0.000 | 0.267 | 0.449 |
|  | HE | 0.752 | 0.485 | 0.471 | 0.325 | 0.673 |
|  | P | < 0.001 | 0.212 | 0.004 | 0.307 | 0.007 |
| OMO361 | NA | 12 | 16 | - | - | 3 |
|  | HO | 0.146 | 0.227 | - | - | 0.000 |
|  | HE | 0.716 | 0.771 | - | - | 0.118 |
|  | P | < 0.001 | < 0.001 | - | - | < 0.001 |
| OMO391 | NA | 11 | 11 | 2 | 6 | 8 |
|  | HO | 0.608 | 0.588 | 0.111 | 0.800 | 0.592 |
|  | HE | 0.779 | 0.703 | 0.111 | 0.773 | 0.679 |
|  | P | < 0.001 | < 0.001 | 1.000 | 0.038 | 0.046 |
| OMO392 | NA | 11 | 6 | 3 | 4 | 3 |
|  | HO | 0.348 | 0.529 | 0.000 | 0.500 | 0.245 |
|  | HE | 0.665 | 0.745 | 0.601 | 0.580 | 0.223 |
|  | P | < 0.001 | < 0.001 | 0.000 | 0.342 | 1.000 |
| OMO397 | NA | 10 | 13 | 2 | 7 | 10 |
|  | HO | 0.506 | 0.765 | 0.111 | 0.567 | 0.755 |
|  | HE | 0.709 | 0.865 | 0.111 | 0.802 | 0.819 |
|  | P | < 0.001 | 0.013 | 1.000 | 0.029 | 0.624 |

**Supplementary Information Table 3. Results of Structure assignments of reference and simulated hybrid data.**

|  | Number assigned to each category | | | |  |  | Probability of assignment of individual to group (mean and range) | | |
| --- | --- | --- | --- | --- | --- | --- | --- | --- | --- |
| Sample | *O. niloticus* | *O. urolepis* | *O. leucostictus* | Hybrid | Total |  | *O. niloticus* | *O. urolepis* | *O. leucostictus* |
|  |  |  |  |  |  |  |  |  |  |
| *O. urolepis* (OU, real) | 0 | 49 | 0 | 0 | 49 |  | 0.048 (0.152-0.023) | 0.925 (0.962-0.83) | 0.025 (0.092-0.013) |
| *O. niloticus* (ON, real) | 30 | 0 | 0 | 0 | 30 |  | 0.937 (0.961-0.834) | 0.036 (0.104-0.024) | 0.026 (0.074-0.012) |
| *O. leucostictus* (OL, real) | 0 | 0 | 9 | 0 | 9 |  | 0.027 (0.044-0.019) | 0.012 (0.019-0.011) | 0.959 (0.97-0.937) |
|  |  |  |  |  |  |  |  |  |  |
| F1 ON x OL | 0 | 0 | 0 | 100 | 100 |  | 0.481 (0.715-0.359) | 0.021(0.061-0.012) | 0.496 (0.622-0.258) |
| F1 OL x OU | 0 | 0 | 0 | 100 | 100 |  | 0.045 (0.121-0.02) | 0.464 (0.581-0.339) | 0.489 (0.624-0.363) |
| F1 ON x OU | 0 | 0 | 0 | 100 | 100 |  | 0.466 (0.679-0.274) | 0.500 (0.684-0.29) | 0.033 (0.111-0.013) |
|  |  |  |  |  |  |  |  |  |  |
| F2 ON x OL | 0 | 0 | 0 | 100 | 100 |  | 0.476 (0.796-0.210) | 0.021 (0.06-0.012) | 0.501 (0.775-0.166) |
| F2 OL x OU | 0 | 0 | 0 | 100 | 100 |  | 0.048 (0.129-0.016) | 0.503 (0.784-0.253) | 0.448 (0.711-0.19) |
| F2 ON x OU | 0 | 0 | 0 | 100 | 100 |  | 0.470 (0.694-0.194) | 0.494 (0.75-0.273) | 0.034 (0.162-0.011) |
|  |  |  |  |  |  |  |  |  |  |
| Backcross OL x (F1 ON x OL) | 0 | 0 | 23 | 77 | 100 |  | 0.244 (0.464-0.021) | 0.017 (0.059-0.012) | 0.738 (0.966-0.52) |
| Backcross OL x (F1 OU x OL) | 0 | 0 | 13 | 87 | 100 |  | 0.041 (0.150-0.019) | 0.234 (0.409-0.09) | 0.724 (0.875-0.552) |
| Backcross ON x (F1 ON x OL) | 25 | 0 | 0 | 75 | 100 |  | 0.742 (0.915-0.54) | 0.035 (0.096-0.012) | 0.222 (0.438-0.058) |
| Backcross ON x (F1 ON x OU) | 18 | 0 | 0 | 82 | 100 |  | 0.728 (0.918-0.466) | 0.243 (0.506-0.068) | 0.027 (0.121-0.012) |
| Backcross OU x (F1 OU x OL) | 0 | 22 | 0 | 78 | 100 |  | 0.053 (0.164-0.025) | 0.737 (0.871-0.577) | 0.208 (0.388-0.054) |
| Backcross OU x (F1 OU x ON) | 0 | 24 | 0 | 76 | 100 |  | 0.215 (0.399-0.06) | 0.753 (0.91-0.546) | 0.031 (0.133-0.012) |
|  |  |  |  |  |  |  |  |  |  |

**Supplementary Information Figure 1**

**SI Fig. 1.** The 25 landmark locations used for quantifying geometric morphometric variation, following Genner *et al*. (2007).

**SI cited literature.**

Genner MJ, Nichols P, Carvalho GR, Robinson RL, Shaw PW, Turner GF (2007) Reproductive isolation among deep-water cichlid fishes of Lake Malawi differing in monochromatic male breeding dress. Mol Ecol 16: 651-662.

Liu F, Sun F, Li J, Xia JH, Lin G, Tu RJ, Yue GH (2013) A microsatellite-based linkage map of salt tolerant tilapia (*Oreochromis mossambicus* x *Oreochromis* spp.) and mapping of sex-determining loci. BMC Genomics 14: 58.

Saju JM, Lee WJ, Orban L (2010) Characterization of nine novel microsatellites isolated from Mozambique tilapia, *Oreochromis mossambicus*. Cons Genet Res 2: 385-387.
